# The effects of *Levilactobacillus brevis* on the physiological parameters and gut microbiota composition of rats subjected to desynchronosis

**DOI:** 10.1186/s12934-021-01716-x

**Published:** 2021-12-20

**Authors:** Evgenii I. Olekhnovich, Ekaterina G. Batotsyrenova, Roman A. Yunes, Vadim A. Kashuro, Elena U. Poluektova, Vladimir A. Veselovsky, Elena N. Ilina, Valeriy N. Danilenko, Ksenia M. Klimina

**Affiliations:** 1 Department of Molecular Biology and Genetics, Federal Research and Clinical Center of Physical-Chemical Medicine of Federal Medical Biological Agency, Moscow, 119435 Russia; 2 Department of Genetics and Biotechnology, Vavilov Institute of General Genetics Russian Academy of Sciences, Moscow, 119991 Russia; 3 Saint Petersburg State Pediatric Medical University, 2 Litovskaya str., St. Petersburg, 194100 Russia; 4 Golikov Research Center of Toxicology Under Federal Medical Biological Agency, 1 Bekhtereva str., St. Petersburg, 192019 Russia

**Keywords:** Biological rhythms, Desynchronosis, *Levilactobacillus*, Metagenomes, Circadian clock, Catecholamines, Gut microbiota, Antioxidant system

## Abstract

**Background:**

All living organisms have developed during evolution complex time-keeping biological clocks that allowed them to stay attuned to their environments. Circadian rhythms cycle on a near 24 h clock. These encompass a variety of changes in the body ranging from blood hormone levels to metabolism, to the gut microbiota composition and others. The gut microbiota, in return, influences the host stress response and the physiological changes associated with it, which makes it an important determinant of health. Lactobacilli are traditionally consumed for their prophylactic and therapeutic benefits against various diseases, namely, the inflammatory bowel syndrome, and even emerged recently as promising psychobiotics. However, the potential role of lactobacilli in the normalization of circadian rhythms has not been addressed.

**Results:**

Two-month-old male rats were randomly divided into three groups and housed under three different light/dark cycles for three months: natural light, constant light and constant darkness. The strain *Levilactobacillus brevis* 47f was administered to rats at a dose of 0.5 ml per rat for one month and The rats were observed for the following two months. As a result, we identified the biomarkers associated with intake of *L. brevis *47f. Changing the light regime for three months depleted the reserves of the main buffer in the cell—reduced glutathione. Intake of *L. brevis* 47f for 30 days restored cellular reserves of reduced glutathione and promoted redox balance. Our results indicate that the levels of urinary catecholamines correlated with light/dark cycles and were influenced by intake of *L. brevis* 47f. The gut microbiota of rats was also influenced by these factors. *L. brevis* 47f intake was associated with an increase in the relative abundance of *Faecalibacterium* and *Roseburia* and a decrease in the relative abundance of *Prevotella* and *Bacteroides*.

**Conclusions:**

The results of this study show that oral administration of *L. brevis* 47f, for one month, to rats housed under abnormal lightning conditions (constant light or constant darkness) normalized their physiological parameters and promoted the gut microbiome's balance.

**Supplementary Information:**

The online version contains supplementary material available at 10.1186/s12934-021-01716-x.

## Background

The oscillations of biological systems and subsystems with a 24-h periodicity are called circadian rhythms (CR). These are entrained by external signals such as light, temperature, feeding, mental or physical stress. Organs such as the liver and the gut possess their own so-termed peripheral clocks, which are normally regulated by the master pacemaker located in the hypothalamus and known as the suprachiasmatic nucleus located in the hypothalamus [1].

CRs are primarily controlled by the core molecular clock, which modulates the activity of transcription factors that regulate the expression of clock-controlled genes found within most host cells [2]. Disruption of light–dark cycles experienced by rotating shift workers, for example, can lead to the desynchronization of the organism's molecular clocks. Eating patterns have also been shown to disrupt CRs as well as the gut microbiota's (GM) composition [3].

Over the last few years, the question of the GM's involvement in this intricate relationship has been addressed in many studies [4–6]. Unsurprisingly, the GM composition fluctuates throughout the day in a pattern that coincides with a person's food intake/fasting cycle. Thus, while the GM rhythmicity is dictated by the organism's routine, the resulting circadian pattern of vitamin production in the gut reinforces the physiological cycle and forms a balancing loop [7]. Aberrant lighting conditions in the form of constant darkness or constant light cause changes in the GM composition in mice through the sensory information received by the eye retina [8, 9]. This was taken a step further in the study of Thaiss et al. by showing that not only the GM composition depends heavily on the eating routine and the light–dark cycles, these changes lead to obesity that is transferred to other that can be transferred to other mice along with the aberrant microbiota. [10]. This implies a causal role of the GM in conveying signals to the brain. Today, it is beyond doubt that desynchronization of the body's internal clock is at the heart of many diseases: altered immune responses [11], multiple chronic inflammation-associated diseases, including irritable bowel syndrome and inflammatory bowel disease, bacterial vaginosis [2, 12, 13], sleep disorders, the metabolic syndrome, digestive disorders and possibly even coronary health [14–16].

Mimicry of jet lag, for instance, in mice, triggers changes in the GM similar to those seen in humans [10]. Sleep fragmentation in rats also alters the GM in ways correlative with mean arterial pressure, which again emphasizes the interconnectivity between CRs, sleep and the GM composition [17].

In a small sample study that compared the GM composition of night shift workers to that of day shift workers revealed distinctive microbial patterns between the two groups. Particularly, the GM of night shift workers was marked by lower abundance of *Bacteroidetes* and higher abundance of *Firmicutes* and *Actinobacteria*. On the genus and species levels, *Faecalibacterium* spp. were associated with normal CRs while high abundance of *Dorea longicatena* and *D.  formicigenerans* was found to be characteristic of night shift workers [4].

The study of the intricate relationship between metabolism and CRs remains a major challenge for chronobiology. A thorough understanding of these interactions will prompt the discovery of alternative ways of both pharmacological and non-pharmacological treatment of CR disorders related to travel across multiple time zones and shift labor in the extreme conditions of territories inside the Arctic circle.

Today the main drugs used for the correction of CRs as a preventive measure against further deteriorations both at the cellular level and at the level of the central nervous system are: melatonin and its analogues (agomelatine, tasimelteon and others); neuropeptides and neuroprotective agents; nootropic drugs and regulatory peptides. Another important category is that of compounds regulating energy metabolism (derivatives of metabolites of the Krebs cycle), which are known to enhance performance in the workplace in extreme conditions.

Probiotic intervention is an innovative approach for the normalization of CRs. This has been evidenced by studies showing a positive restorative effect of probiotics and prebiotics on sleep quality [18, 19] and circadian desynchrony [20–23]. Since GABA's central role for sleep has been well documented, its implication in this relationship cannot be completely dismissed.

In a previous paper, we investigated rats subjected to normal light–dark cycles, darkness and constant lighting for one and three months. Changing the lighting conditions led to changes in almost all the studied physiological parameters: urinary catecholamine (CA) levels, indicators of lipid peroxidation and antioxidant activity in the blood; protein levels of BMAL1, CLOCK and THRA in the hypothalamus; composition and functional activity of the GM [9]. In this study, we investigated the impact of different lighting conditions (12-h day-night cycles, constant light, constant darkness) on the physiological parameters and GM composition of untreated rats and rats receiving the strain *Levilactobacillus brevis* 47f – a potential probiotic strain capable of GABA production [24]. The strain also possesses antioxidant properties according to a series of in vitro and in vivo studies. *L. brevis* 47f was initially tested for antioxidant properties using the test system *E. coli* MG1655 carrying plasmids encoding luminescent biosensors pSoxS-lux and pKatG-lux. The cell-free culture supernatant of the strain proved effective in countering oxidative stress triggered by superoxide anion and hydrogen peroxide [25]. *L. brevis* 47f also stimulated local anti-inflammatory activity and increased total antioxidant levels in the blood and intestines of BALB/c mice experiencing 5-fluorouracil-induced mucositis [26].

## Results

### Differential assay of urinary CAs

Analysis of the concentration of CAs in the rat urine revealed several changes related to the lighting conditions. Comparing the urinary CA levels between animals subjected to constant light or darkness to those living under alternating night and day cycles during one month uncovered an decrease in dopamine (DA) levels. More specifically, the constant light (LL1) and constant darkness (DD1) groups exhibited a 36.9% and 25.7% decrease, respectively. Urinary DA levels dropped by 28.9% in rats treated with *L. brevis* 47f as compared to non-treated rats, both held in normal day/night cycles (LD1) (Table 1).

**Table 1:** Concentration of urinary CAs of rats after one month

Light regimes	Normal day/night cycles (LD1)		Constant light (LL1)		Constant darkness (DD1)	
Indicator	Without *L. brevis* 47f	*L. brevis* 47f	Without *L. brevis* 47f	*L. brevis* 47f	Without *L. brevis* 47f	*L. brevis* 47f
Dopamine, µg/l	285.7 ± 15.2	202.9 ± 10.3*	180.1 ± 4.9**	200.5 ± 3.8*	212,1 ± 3.5	203.9 ± 7.9
Noradrenalin, µg/l	70.1 ± 9.1	73.2 ± 8.5	75.0 ± 7.4	60.3 ± 8.2	34,6 ± 10.8**	50.7 ± 4.6*#
Adrenaline, µg/l	26.3 ± 1.3	32.1 ± 1.2*	25.5 ± 2.1	26.7 ± 1.4	30,5 ± 2.1	32.7 ± 1.6

*significant differences compared to control in each group p ≤ 0.05

**Compared to LD1 p ≤ 0.05

^#^Compared to LD1 *L*. *brevis* 47f p ≤ 0.05

The rats that were held in constant light for one month while receiving the strain *L. brevis* 47f exhibited an increase in DA levels by 11.3% as compared to non-treated rats held in the same conditions. Moreover, DA urinary levels of rats treated with *L. brevis* 47f were comparable to those rats held under normal day/night cycles.

Noradrenalin (NA) urinary levels also diminished by 50.5% in light-deprived rats as compared to the control group held under normal lighting conditions.

Rats held in constant darkness and receiving *L. brevis* 47f during the first 30 days of the experiment demonstrated significantly higher levels of urinary NA levels exceeding non-treated rats by 46.5%. However, when compared with the group LD1 receiving *L. brevis* 47f, NA urinary levels of rats held in constant darkness and treated with *L. brevis* 47f were lower by 30.7%.

Our results also show that gavaging rats held in constant darkness with *L. brevis* 47f, for 30 days, restored their adrenaline (A) levels to those observed in rats under normal lighting conditions receiving *L. brevis* 47f. Housing rats under abnormal lighting conditions for additional two months disrupted further their urinary CA levels. In particular, urinary DA levels dropped by 73.8% in rats housed in constantly illuminated cages (LL3) and increased by 34.9% in rats held in constant darkness (DD3) (Table 2).

**Table 2:** Concentration of urinary CAs of rats after three months

Light regimes	Normal day/night cycles LD3		Constant light LL3		Constant darkness DD3	
Indicator	Without *L. brevis* 47f	*L. brevis* 47f	Without *L. brevis* 47f	*L. brevis* 47f	Without *L. brevis* 47f	*L. brevis* 47f
Dopamine, µg/l	330.1 ± 12.2	790.3 ± 15.4*	86.4 ± 12.4**	312.2 ± 12.5*#	445.6 ± 15.4**	388.4 ± 14.3*#
Noradrenalin, µg/l	207.0 ± 14.5	232.4 ± 12.5	85.7 ± 13.3**	100.4 ± 8.0#	162.8 ± 11.2**	114.3 ± 10.4*#
Adrenaline, µg/l	79.1 ± 1.4	97.6 ± 10.8*	29.8 ± 1.3**	13.4 ± 1.2*#	108.7 ± 8.4**	20.2 ± 1.1*#

*significant differences compared to control in each group, p ≤ 0.05

**Compared to LD3 p ≤ 0,05

^#^Compared to LD3 *L*. *brevis* 47f p ≤ 0,05

Moreover, comparison of urinary DA levels, after three months, between rats receiving *L. brevis* 47f and non-treated rats, both housed in normal day/night cycles, revealed a 2.4-fold increase in the former group. Treatment with *L. brevis* 47f also affected urinary DA levels of rats held in abnormal lighting conditions as compared to non-treated rats, yielding a 3.6-fold increase in the LL3 groups and 12.8-fold decrease in the DD3 group. It is noteworthy that urinary DA levels of the *L. brevis* 47f group (LL3 and DD3) were comparable to those of the LD3 group.

Urinary NA levels of the LL3 group dropped by 58.6% compared to the LD3 group. After three months, urinary NA levels in the LL3 and DD3 groups that received *L. brevis* 47f increased by 17.1% and dropped by 29.7%, respectively, as compared to non-treated rats. Urinary NA levels in LL3 and DD3 receiving *L. brevis* 47f dropped by 56.7 and 50.8%, respectively, when compared with LD3 that received *L. brevis* 47f.

Urinary A levels were also altered in rats after three months, dropping by 62.3% and 37.4% in LL3 and DD3, respectively, as compared to LD3. Treatment of rats with *L. brevis* 47f led to a decrease in urinary A levels by 55.0% and 81.4%, in LL3 and DD3, respectively, as compared to non-treated rats. Comparing LL3 and DD3 to LD3Lbrevis revealed an 86.2% and 79.3% drop, respectively.

### Indicators of lipid peroxidation and antioxidant activity in the blood

Rats that lived in constant light or in constant darkness, for one month, demonstrated a 3.2-fold and 2.3-fold increase in the level of primary products of lipid peroxidation (conjugated dienes (CD) in erythrocyte hemolysates), respectively. Also, after one month, the rats receiving *L. brevis* 47f in the groups LL1 and DD1 exhibited lower levels of CD amounting to a decrease by 32.8% and 58.5%, respectively, as compared to the corresponding groups of non-treated rats. There were no significant differences, however, in the level of CD between the groups DD1 receiving *L. brevis* 47f and the non-treated LD1 group (Table 3).

**Table 3:** Indicators of antioxidant activity and lipid peroxidation in the erythrocytes of rats after one month

Light regimes	Normal day/night cycles (LD1)		Constant light (LL1)		Constant darkness (DD1)	
Indicator	Without *L. brevis* 47f	*L. brevis* 47f	Without *L. brevis* 47f	*L. brevis* 47f	Without *L. brevis* 47f	*L. brevis* 47f
Reduced Glutathione, µM/gHb	9.8 ± 0.4	10.4 ± 0.3	7.6 ± 0.5	10.6 ± 0.4*	9.6 ± 0.4	12.8 ± 0.3*#
Malondialdehyde, µM/gHb	20.1 ± 2.0	16.7 ± 1.3	20.8 ± 0.3	19.5 ± 0.2	21.9 ± 2.8	17.6 ± 2.0
Conjugated dienes, µM/gHb	1.8 ± 0.1	1.1 ± 0.1	5.8 ± 0.5**	3.9 ± 0.2*#	4.1 ± 0.1**	1.7 ± 0.2*#
Superoxide dismutase, U/gHb	3562.6 ± 140.3	4365.9 ± 123.0*	2455.0 ± 174.0**	4564.4 ± 110.5*	2689.4 ± 218.0	3849.4 ± 117.2*#
Glutathione S-transferase, U/gHb	145.0 ± 1.5	120.1 ± 1.6*	105.2 ± 5.5**	129.1 ± 9.3	99.7 ± 3.7**	115.0 ± 3.7
Glutathione peroxidase, U/gHb	27.1 ± 0.4	30.3 ± 0.6	36.4 ± 1.2**	39.5 ± 1.7#	44.5 ± 0.7**	40.0 ± 1.1#
Glucose-6-phosphate dehydrogenas, U/gHb	8.1 ± 0.3	9.7 ± 0.3*	5.9 ± 0.2**	8.6 ± 0.3*#	7.2 ± 0.3**	9.2 ± 0.3*

*significant differences compared to control in each group p ≤ 0.05

**Compared to LD1 p ≤ 0.05

^#^Compared to LD1 *L*. *brevis* 47f p ≤ 0.05

The superoxide dismutase (SOD) activity in erythrocyte hemolysates was significantly higher in rats treated with *L. brevis* 47f. SOD activity was mostly pronounced in the LL1 group, exceeding its levels in non-treated rats held in the same conditions by 85.9%.

At the same time, the glutathione S-transferase (GST) activity significantly dropped in rats held under abnormal lighting conditions. More specifically, GST activity was lower by 27.5% and 31.2% in the LL1 and DD1 groups as compared to LD1. Treatment of rats with *L. brevis* 47f restored GST activity to the levels of the group LD1 *L*. *brevis*. Moreover, treatment of rats with *L. brevis* 47f boosted GST activity in the groups LL1 and DD1 by 30.4% and 32.0%, respectively, as compared to the LD1 *L*.*brevis* group.

The activity of glucose-6-phosphate dehydrogenase (G6PD) in erythrocyte hemolysates significantly diminished in rats kept constantly in darkness or in illuminated cages. G6PD activity dropped by 27.2% and 11.1% in the non-treated LL1 and DD1 groups, respectively, as compared to the non-treated LD1 group. Treatment of rats with *L. brevis* 47f increased G6PD activity by 19.7%, 45.7% and 27.7% in the groups LL1, DD1 and LD1, respectively, as compared to non-treated rats kept in the same conditions. These results indicate that treatment with *L. brevis* 47f restored G6PD levels.

Constant light contributed to a drop in the cellular reserves of the buffer reduced glutathione (RG). Rats that have received *L. brevis* 47f exhibited normal levels of RG in both constant light and constant darkness groups. RG levels in the LD1 *L*. *brevis* group were higher by 23.0% as compared to the non-treated LD1 group.

Extended exposure to constant light or darkness up to three months maintained increased levels of CD in erythrocyte hemolysates, which surpassed the LD3 group by 78.9% in the LL3 and 2.7-fold in the DD3. Comparison of treated rats to non-treated rats held in the same conditions revealed lower levels of CD in the treated rats: CD levels dropped by 38.2% and 43.1% in constant light and constant darkness groups, respectively. Comparison of rats receiving *L. brevis* 47f to each other revealed a 23.5% and 70.5% increase in the groups LL3 and DD3, respectively, as compared to LD3 (Table 4).

**Table 4:** Indicators of antioxidant activity and lipid peroxidation in the erythrocytes of rats after three months

Light regimes	Normal day/night cycles LD3		Constant light LL3		Constant darkness DD3	
Indicator	Without *L. brevis* 47f	*L. brevis* 47f (LD1_Lbr_)	Without *L. brevis* 47f	*L. brevis* 47f (LL1 _Lbr_)	Without *L. brevis* 47f	*L. brevis* 47f (DD1 _Lbr_)
Reduced Glutathione, µM/gHb	10.3 ± 0.6	12.4 ± 0.2*	8.1 ± 0.3**	10.7 ± 0.2*#	9.5 ± 0.2	12.2 ± 0.2*
Malondialdehyde, µM/gHb	21.0 ± 1.9	13.5 ± 0.4*	19.6 ± 0.5	15.2 ± 0.4*#	22.4 ± 0.4	15.9 ± 0.4*#
Conjugated dienes, µM/gHb	1.9 ± 0.2	1.7 ± 0.1	3.4 ± 0.4**	2.1 ± 0.1*#	5.1 ± 0.2**	2.9 ± 0.2*#
Superoxide dismutase, U/gHb	4104.6 ± 141.1	3749.0 ± 127.4	4880.6 ± 119.4	3338.6 ± 105.5*	4243.5 ± 128.0	3934.5 ± 117.4
Glutathione S-transferase, U/gHb	140.6 ± 18.2	152.0 ± 18.7	197.2 ± 14.4**	130.5 ± 15.9*	251.8 ± 15.3**	162.4 ± 12.0*
Glutathione peroxidase, U/gHb	35.9 ± 0.4	32.3 ± 0.8	40.2 ± 0.7	35.0 ± 0.4*	46.7 ± 1.7	37.5 ± 1.4#
Glucose-6-phosphate dehydrogenase, U/gHb	11.7 ± 1.5	10.8 ± 1.2	8.5 ± 1.3**	10.4 ± 1.2*	7.1 ± 1.5**	11.1 ± 0.6*

*significant differences compared to control in each group p ≤ 0.05

**Compared to LD1 p ≤ 0.05

^#^Compared to LD3 *L*. *brevis* 47f p ≤ 0.05

GST levels in erythrocyte hemolysates of groups LL3 and DD3 were higher by 40.7% and 79.2% than LD3. There was no significant difference, however, between treated and non-treated rats, held under normal conditions. Interestingly, glutathione peroxidase (GP) levels were higher by 16% in the DD3Lbrevis group than the LD3Lbrevis group.

After three months of exposure to abnormal lighting conditions, the rats exhibited a significant decline in G6PD levels in erythrocyte hemolysates. More specifically, the groups LL3 and DD3 exhibited a 27.3% and 39.3% decrease, respectively, as compared to LD3. Rats that were treated with *L. brevis* 47f for 30 days demonstrated higher G6PD levels reaching 22.3% and 56.3% increase in the groups LL3 and DD3, respectively, as compared to non-treated rats. These results suggest that treating rats with *L. brevis* 47f restored their G6PD levels back to normal. While cellular stores of RG were depleted in non-treated rats housed under abnormal lighting conditions for 30 days, in treated rats, they stayed within the normal range.

### Analysis of the GM composition of rats

**Fig. 1:**
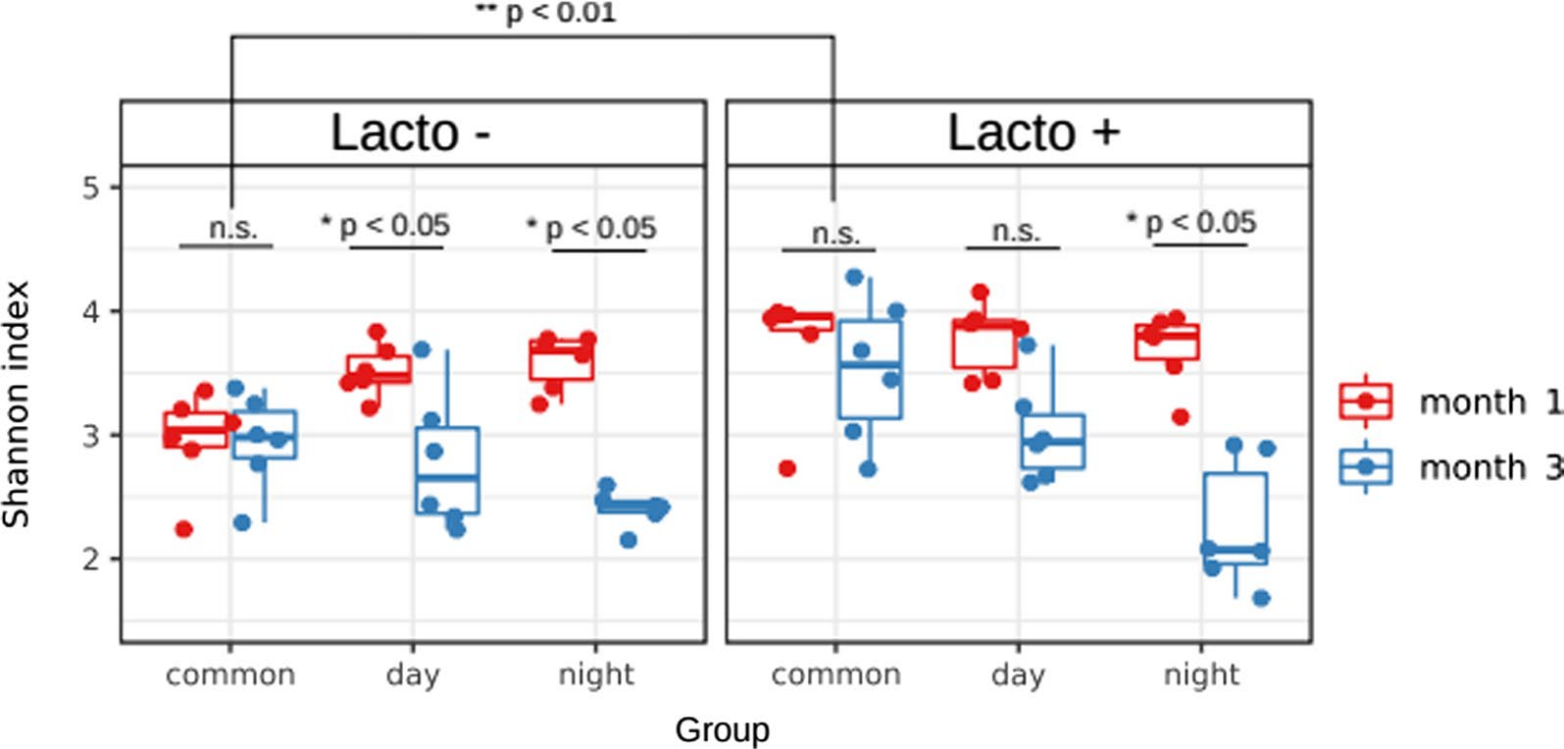
Alpha-diversity distribution of stool samples across all experimental groups at different time points. Statistical assessment was performed using Wilcoxon rank-sum and Wilcoxon signed-rank tests

Sequencing of fecal DNA yielded 10,919 ± 6,180 250-long b.p. paired preprocessed reads per sample. The DADA2 pipeline and SILVA database allowed us to identify a total of 409 amplicon sequence variants (ASV) belonging to eight bacterial phyla and 60 bacterial genera. Analysis of the microbiome richness showed that treating rats with *L. brevis* 47f increased alpha diversity in the normal day/night cycle group but not in the groups experiencing desynchronosis (Fig. 1, Wilcoxon rank-sum test, p < 0.01). Overall, desynchronosis-related stress led to a decrease in the GM alpha diversity of rats regardless of *L. brevis* 47f supplementation (Wilcoxon rank-sum test, p < 0.01)*.* Nevertheless, statistical analysis revealed that the constant light + *L. brevis* 47f group was less prone to a decline in bacterial diversity in comparison with the non-treated rats held in the same conditions (Fig. 1, Wilcoxon signed-rank test, p < 0.05 in full day Lacto- group vs Wilcoxon signed-rank test, p > 0.05 full day Lacto + group).

**Fig. 2:**
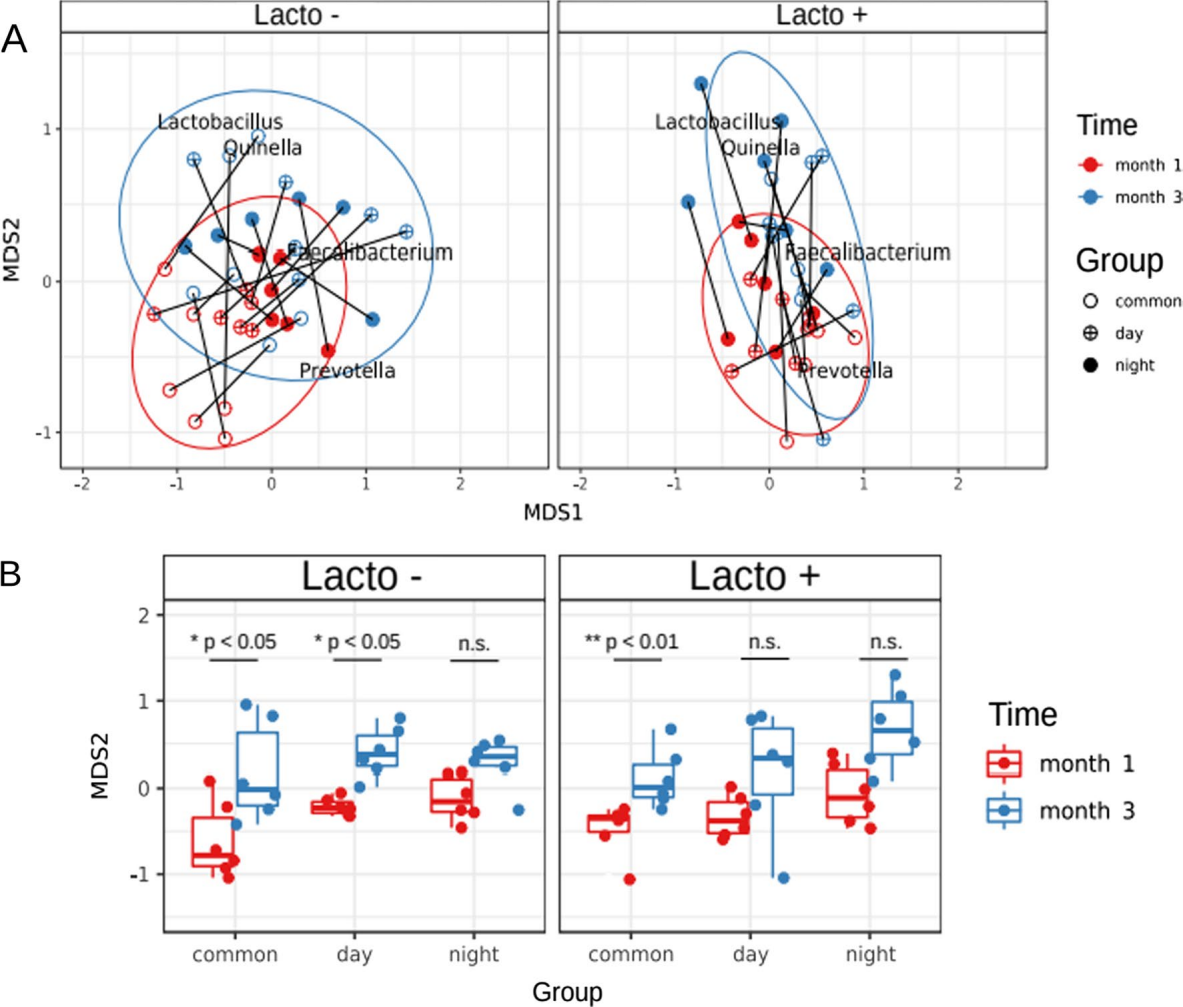
Analysis of the GM composition of experimental groups. **A** Non-metric multidimensional scaling biplot of taxonomic profiles of mice’s stool samples using 16S rRNA gene sequencing and the Bray–Curtis dissimilarity index. **B** MDS2 component distribution across experimental groups and time. Statistical significant differences were assessed using the Wilcoxon signed-rank test

Non-metric multidimensional scaling visualisation using the Bray–Curtis dissimilarity index indicated that a shift in bacterial composition occurs over time (see Fig. 2A). The general tendency to Y-axis (MDS2 component) shifts from *Prevotella* to *Lactobacillus* and *Quinella* dominance (Wilcoxon rank-sum test, p < 0.01). Interestingly, this shift was common to all experimental groups and was not associated with *L. brevis* 47f intake. However, the intensity of these changes differed between the experimental groups (see Fig. 2B).

We used the Songbird software to identify biomarkers of desynchronosis in the GM. The Songbird software generates a file containing differentials as its primary output. These describe log-fold changes in features with respect to certain field(s) in sample metadata. The most important aspect of these differentials is their rankings, which are obtained by sorting a column of differentials from lowest to highest. These rankings inform us of the relative associations of features with a given covariate [27].

**Fig. 3:**
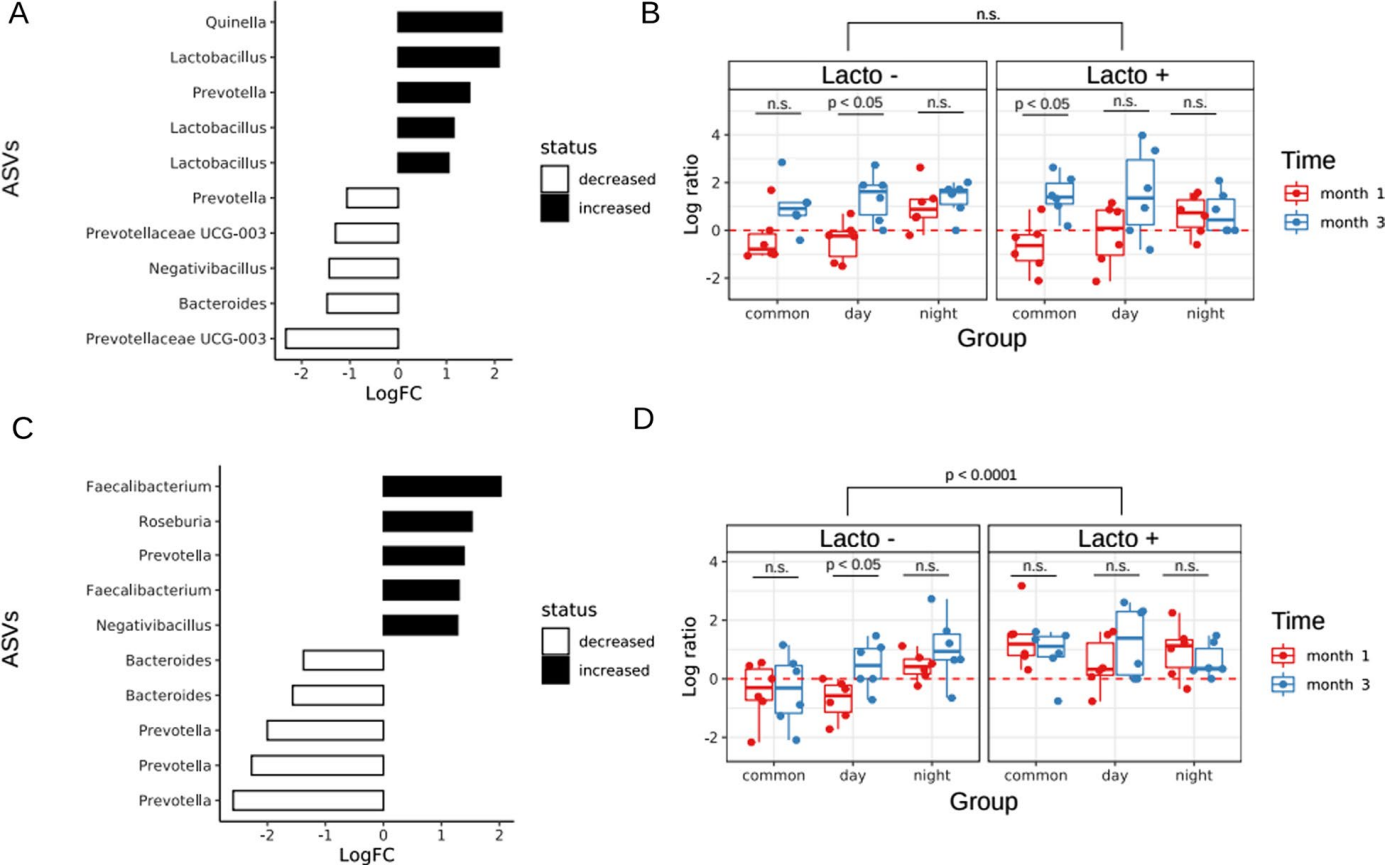
Differences of rats GM taxonomic composition between experimental groups. **A**, **C** The Songbird taxonomy differentials analysis via ‘rank plots’. Y-axis shows microbial taxonomy, X-axis shows differentials, which describe the log-fold change in features with respect to time points (**A**, **C**) *L. brevis* 47f intake. **B**, **D** Qurro ‘sample plots’ describes feature log-ratios in the context of feature rankings presented in **A**, **C**. **A, B** Time-dependent differences in GM composition. **C**, **D** Differences in GM composition in dependence of *L. brevis* 47f intake

Analysis of the biomarkers of desynchronosis in the GM confirmed that the changes in the GM composition were independent of *L. brevis* 47f intake (see Fig. 3A). We found that in the experimental groups, the relative abundance of *Lactobacillus* spp. and *Quinella* spp. increased whereas *Prevotellaceae* UCG-003 and *Bacteroides* spp. decreased over time. *Prevotella* spp. showed conflicting results. Analysis of log-ratios in the context of feature rankings and sample metadata was carried out using the Qurro tool [23]. Log-ratios of the selected taxonomic features associated with time across samples are provided on Fig. 3B (Wilcoxon rank-sum test, p < 0.01). It is important to point out that the intensity of change between the experimental group varied significantly (see Fig. 3B). The Songbird approach allowed us to identify biomarkers associated with *L. brevis* 47f intake. *L. brevis* 47f intake was associated with higher abundance of *Faecalibacterium* and *Roseburia* and lower abundance of *Prevotella* and *Bacteroides* (see Fig. 3C). Log-ratios analysis of biomarkers showed that Lacto- constant darkness group at time points one (DD1) and three (DD3) months and Lacto- constant light group at time point three months (LL3) were associated with higher abundance of *Faecalibacterium* and *Roseburia* in comparison with Lacto- control groups (see Fig. 3C).

## Discussion

Disturbances in the periodicity of light and darkness cycles caused significant changes in experimental rats, namely in the levels of urinary CAs, erythrocyte hemolysate levels of antioxidant proteins and markers of oxidative stress as well as the composition and diversity of the GM. These changes were much more dramatic three months into the experiment than after one month. Oral administration of the bacterial strain *L. brevis* 47f normalized most of the studied parameters, more specifically, it increased the levels of RG and G6PD in erythrocyte hemolysates, decreased of MA and CD, changed the composition of the GM by tipping the balance towards higher abundance of *Faecalibacterium* and *Roseburia* and lower abundance of *Prevotella* and *Bacteroides*.

CAs are biogenic amines that are synthesized from the conditionally essential amino acid tyrosine. CAs are formed in the brain and other tissues of the nervous system where they act as neurotransmitters. Peripheral DA is synthesized by chromaffin cells of the adrenal medulla, intestinal epithelial cells and by kidney proximal tubules. Peripheral NA and A are synthesized and stored inside secretory vesicles of the adrenal glands in very high concentrations until they are released through exocytosis. Peripheral CAs are suspected to play a role in mental and noradrenergic modulation. Moreover, the GM is known to actively synthesize CAs, thereby potentially affecting the psycho-emotional state of the host organism. The functional proximity of CAs to the sympathetic nervous system also suggests that they are not only involved in activating the body's defense systems against acute stress, but perhaps they can be seen as an indicator of the body's adaptive reserves when it is subjected to chronic stress [28–31].

Moreover, while DA levels are elevated in the retina's cells of animals subjected to constant light, constant darkness elicits opposite effects caused by melatonin [32, 33]. Our data demonstrate that light also plays an important role in regulating urinary DA levels. Urinary DA levels dropped in rats subjected to changing lighting regimes. Interestingly, treating rats with *L. brevis* 47f for a 30-day period restituted urinary DA levels in the constant light groups to levels comparable to those of the LD3 group. The same effect was seen in the constant darkness group.

DA levels in the tissues, the blood plasma, and the urine are strongly aligned with circadian fluctuations. NA and A levels are also tied to CRs due to their active regulatory role of carbohydrate-lipid metabolism, and thus their concentration in the blood plasma and the tissues both depend on the intensity of energy metabolism. In our previous study, we demonstrated that light deprivation disrupts the antioxidant balance and energy metabolism in cells. NA is also associated with the hormone melatonin. The moment darkness settles in, a nerve impulse triggers the release of NA, which in turn activates the retino-hypothalamus-pineal gland thereby leading to an increase in the expression of α1 and β1 adrenergic receptors in the pineal gland and stimulating the secretion of melatonin. Interestingly, the secretion of melatonin is not limited to the nervous system, it is secreted in the GIT, the respiratory tract, the pancreas, the adrenal glands, the liver, and the kidneys [34, 35].

In our study, while NA urinary levels of rats held constantly in darkness (DD1) dropped significantly, they were much higher in the rats that received *L. brevis* 47f and were held in similar conditions for 30 days. Feeding rats the strain *L. brevis* 47f for a period of 30 days had the opposite inhibiting effect in rats housed under abnormal lighting conditions. Constant light or darkness are regarded as chronic stressors that are likely to exacerbate the rats emotional state.

In recent years, many studies established a link between CRs and redox systems. The existence of a relationship between these two systems is hardly surprising since most organisms exhibit daily rhythmic fluctuations in energy consumption and motor activity. These fluctuations are a result of external influences such as the consumption of food and internal factors such as the secretion of biologically active compounds by the intestinal microbiome. Moreover, circadian clock proteins control the cell's redox status and the abundance of cofactors required for redox reactions. At the same time, cellular redox homeostasis also affects the transcriptional oscillator via redox-sensitive transcription factors and enzymes [36, 37].

Thus, studying the relationship between CRs, the GM composition (qualitatively and quantitatively) and redox systems is an up-to-date fundamental task.

Both the scientific literature and our previous studies show that chronic abnormal lighting conditions cause tissue hypoxia, which leads to the formation of ROS. Active oxygen radicals damage the lipids of cell membranes by activating free-radical cascades, which contribute to the intensification of lipid peroxidation and the activation of enzymatic and non-enzymatic mechanisms of the cell's antioxidant system. On the other hand, structural changes in erythrocyte membranes affect the diffusion rate of O2 and CO2 through the membrane, a potential factor of tissue hypoxia [38, 39].

Gavaging rats with *L. brevis* 47f for 30 days reduced the concentration of lipid peroxidation end products such as CD and MA. It is possible that this is a result of an increase in the activity of the enzyme SOD that represents the first line of defense against free radicals. *L. brevis* 47f also reduced the activity of another enzyme, GP. *L. brevis* 47f also increased the activity of G6PD, which restores glutathione cellular reserves. In the hexose monophosphate pathway, the enzyme G6PD in erythrocytes catalyzes the formation of NADPH, which is necessary for the restoration of RG levels [40–42].

Members of the genus *Faecalibacterium* were consistently found to be more abundant in our previous study that set out to examine the influence of constant light and constant darkness on the GM composition of rats [9]. Considering the importance of *Faecalibacterium prausnitzii*, the sole known member of the genus, for a healthy GM, we could conjecture that an increase in the abundance of this genus in stressful conditions might compensate for the decline in the diversity of the GM. Interestingly, both *Faecalibacterium* and *Roseburia* are prolific producers of butyrate and other short-chain fatty acids, which are crucial for the integrity of the gut barrier and health as a whole [43]. *Roseburia* was also associated with weight loss in a mouse model of vertical sleeve gastrectomy [44]. Therefore, it is possible that the rise in the abundance of *Roseburia* spp. counters the weight gain characteristic of mice with altered CRs [45]. Contrastingly, *Prevotella* is a diverse genus of gram-negative bacteria that is associated with carbohydrate consumption and inflammation in the gut [46, 47]. Although their function in the gut remains controversial, in the light of what is known about *Prevotella* spp., their decrease is likely to be beneficial. The decrease in the abundance of *Bacteroides* can be seen as a reversal of the results obtained previously [9].

*L. brevis* is a heterofermentative gram-positive organism frequently isolated from milk, cheese, sauerkraut, sour dough, silage, the oral cavity and the intestinal tract of humans [4]. *L. brevis* enjoys GRAS status (Generally Recognized As Safe) on the basis of their widespread historical use in various traditional fermented food products [48]. Strains of *L. brevis* can vary significantly with some possessing antipathogenic [49], antioxidant, immunomodulatory [50], anticarcinogenic [51] and antifungal [52] activity, all considered probiotic properties.

The association between the GM composition and altered CRs was addressed in a small sample study authored by Mortas et al. [4]. It is noteworthy that disrupted CRs are a cause of a decrease in sleep quality and sleep duration. Today, only a few inconsistent studies have examined the effects of sleep restriction on the GM. One of the earliest studies in this field conducted on rats showed that sleep deprivation for a period of 10 days led to intestinal overgrowth of aerobes and facultative anaerobes including several pro-inflammatory and pathogenic species [53].

It is generally accepted that sleep restriction leads to increased food consumption [53, 54]. This is possibly one of the indirect mechanisms underlying the impact of sleep restriction on the GM composition, which falls in line with the findings of Poroyko et al. that the microbial taxa that thrived following sleep fragmentation happen to be those capable of degrading indigestible fibers, such as *Lachnospiraceae* and *Ruminococcaceae* [54]*.*

## Conclusion

The administration of *L. brevis* 47f to rats elicited an increase in the abundance of SCFA-producing bacteria such as *Faecalibacterium* and *Roseburia*, which are both associated with anti-stress and antidepressant effects [55–58]. Perhaps, the GM counters chronic stress by favoring the growth of some of its members that are likely to confer health benefits. At the same time, the more intense the stress level is (for instance, constant light is considered a more potent stressor than constant darkness), the longer it takes the GM to adapt to these conditions. Moreover, *L. brevis* 47f administration to rats for 30 days stabilized the redox status of erythrocyte cells, which is undoubtedly beneficial for the body's adaptation to abnormal lighting conditions in the long run, and reduced the concentration of oxidative stress markers such as CD and MA. In alignment with these findings, *L. brevis* 47f could confer beneficial antistress effects to subjects experiencing desynchronosis.

## Methods

All studies were conducted in accordance with the GSK Policy on care, welfare and treatment of laboratory animals and were approved both by the Institute of Toxicology Federal Medical-Biological Agency of Russia (Bioethics Committee) and by the Veterinary Department of Saint Petersburg.

36 two-month-old male Wistar rats were purchased from the "Rappolovo" nursery in Saint Petersburg and randomly divided into three groups: a control group light:dark (12:12) under led lighting (500 lx), an experimental group under constant-lighting conditions (500 lx), and an experimental group under constant- darkness conditions. The rat's weight changes across the experimental groups are presented in Additional file 1: Figure S1.

The rats were held in standard cages (n = six) at + 21–23 °C and were fed a standard laboratory chow diet. Feces, blood and urine samples were collected after one and three months. The study was carried out in a GLP-accredited laboratory.

### Growth and Lyophilization conditions

The strain *L. brevis* 47f was grown as previously described [26] under anaerobic conditions (10% CO2 atmosphere, Anaerobic System Mark II, HiMedia, Mumbai, India) at + 37 °C in MRS medium (HiMedia). To obtain the final product for biological testing, the bacterial culture was cultivated in a fermenter under anaerobic conditions for 18–20 h until it reached the stationary phase (10^9^ CFU/mL). Then the culture was washed with sterile PBS buffer and transferred to a solution containing 1% gelatin and 10% sucrose, incubated for 24 h at − 20 °C and dried in a 2.5-L Labconco freeze dryer (Labconco, Kansas City, MO, USA) under a pressure of 0.42 mBar and a temperature of − 52 °C for 48 h. Vials were stored at + 4 °C and the viability of the lyophilisates did not change over the course of one year. The lyophilized strain *L. brevis* 47f was dissolved in a saline solution and administered to rats orally at a dose of 0.5 ml per rat for 1 month.

### DNA Extraction and 16S rRNA Amplicon Sequencing

DNA was extracted from feces using the MagNA Pure Compact Nucleic Acid Isolation Kit I (Roche, Germany) according to the manufacturer’s protocol for bacterial DNA. The lysis of bacterial cells was performed using the MagNA Pure Bacteria Lysis Buffer following the recommended protocol for stool samples. The gDNA quantity was determined using the Qubit 2.0 Fluorometer (Invitrogen, USA) following the manufacturer’s protocol. Sequencing of the V3-V4 region of the 16S rRNA gene was performed following 16S Metagenomic Sequencing Library Preparation protocols. (https://support.illumina.com/downloads/16s_metagenomic_sequencing_library_preparation.html). Sequencing was performed using Illumina MiSeq Systems (Illumina, USA) with 2 × 250 bp paired-end runs following the manufacturer's instructions.

### Assessment of CA levels in the urine

Urine was collected from each metabolic individual cage between 11.30 am and 12.30 am. Urinary CAs were separated using a SHIMADZU liquid chromatograph on an Inertsil ODS-EP column (Shimadzu, Japan) with electrochemical detection for measuring concentration. Sample preparation was carried out using a CAs Urine kit (Chromsystems, Germany).

### Lipid peroxidation and antioxidant activity

In order to separate the plasma from the erythrocytes, we centrifuged blood at 3000 rpm and + 4 °C for 10 min. Erythrocytes were washed three times with a cold physiological solution and centrifuged repeatedly. Hemolysis of erythrocytes was performed by adding a 5-mM Tris–HCl buffer, pH 7.6, to the cell suspension at a ratio of 1:9. Blood was then incubated at + 4 °C for 30 min. The hemolysate was used in the subsequent assays in conjunction with the reagent kits supplied by Randox (United Kingdom).

The concentrations of RGs in erythrocyte hemolysate was determined using 5,5′-di-thio-bis(-2-nitrobenzoic) acid (DTNB) by the method of G. L. Ellman (1959) [59]. The activity of GST was determined by the method of W. H. Habig and W. B. Jacoby (1981) [60]. Concentrations of CDs in tissue homogenates and lysed erythrocytes were determined by the method of I. D. Steel (1977) [61]. The concentration of MDA was determined by the method of Uchiyama M. (1978) [62]. Hemoglobin in hemolysates of erythrocytes was determined using standard kits Ecolab (Russia). Blood samples were collected from 11.30 a.m. to 12.30 a.m.

### Data analysis

The blood and urine tests were subjected to statistical analysis using the Statistica 6.0 software. Standard deviation (SD) and standard error of the mean (SEM) were calculated and provided. Normality was checked using the Shapiro–Wilk test. The Wilcoxon rank-sum test was used to test the differences in the collected data between experimental groups.

The data generated by 16S rRNA gene sequencing was processed through the DADA2 pipeline [63] according to published protocols [64] and the SILVA database [65]. Finally, we obtained the phyloseq [66] object, which contains an amplicon sequence variant (ASV) table, a taxonomy table, and a phylogenetic tree. The Songbird [27] and Qurro [67] approaches implemented in the QIIME2 framework [68] were used to identify the biomarkers that vary among experimental groups. Wilcoxon signed rank test was used for additional statistical comparison. Data visualization was performed using ggplot2 [https://ggplot2.tidyverse.org] and vegan [69] libraries implemented for GNU/R [70].

## Supplementary Information

**Additional file 1: Figure S1.** Weight gain by rats from different experimental groups one month after the start of the experiment.

## Data Availability

All data generated or analyzed during this study are included in this published article and its additional files. 16S rRNA gene sequence data (fastq files) were submitted to the NCBI’s SRA (Bioproject: PRJNA760288).

## Abbreviations

LD: Normal day-night cycles
LL: Constant light
DD: Constant darkness
SCN: Superchiasmatic nucleus
GM: Gut microbiota
CRs: Circadian rhythms
CAs: Catecholamines
DA: Dopamine
GST: Glutathione S-transferase
G6PD: Glucose-6-phosphate dehydrogenase
GPx: Glutathione peroxidase
MDA: Malondialdehyde
CD: Conjugated dienes
GPx: Glutathione peroxidase
SOD: Superoxide dismutase
NADPH: Nicotinamide adenine dinucleotide phosphate
GSSG: Glutathione disulfide
GSH: Glutathione
PAS: Per-Arnt-Sim
SDS-PAGE: Sulfate–polyacrylamide gel electrophoresis
RG: Reduced Glutathione
