# Supplementary figures and images for: The effects of Levilactobacillus brevis on the physiological parameters and gut microbiota composition of rats subjected to desynchronosis
Source: Microb Cell Fact. 2021 Dec 20;20:226. doi: 10.1186/s12934-021-01716-x (PMC8686522; doi:10.1186/s12934-021-01716-x)

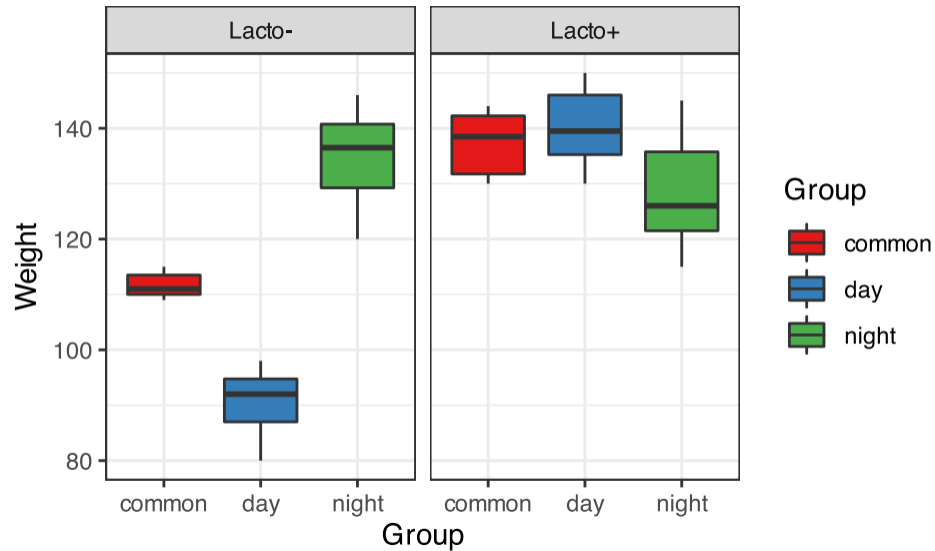

Supplement: Supplementary file 1 — Additional file 1: Figure S1. Weight gain by rats from different experimental groups one month after the start of the experiment. [file 12934_2021_1716_MOESM1_ESM.pdf]
